# Supplementary material for: How nature discovers rare Turing islands: Exploration by common limit cycles
Source: Proc Natl Acad Sci U S A. 2026 Jun 4;123(23):e2536690123. doi: 10.1073/pnas.2536690123 (PMC13250505; doi:10.1073/pnas.2536690123)
Supplement: Supplementary file 1 — Appendix 01 (PDF) [file pnas.2536690123.sapp.pdf]

PNAS

Supporting Information for

How Nature discovers rare Turing islands: exploration by common limit cycles

Seyoon Kim, Antonio Matas-Gil, and Robert G. Endres

Robert G. Endres.
E-mail: r.endres@imperial.ac.uk

This PDF file includes:

Supporting text
Figs. S1 to S13
SI References

Supporting Information Text

Overview

We provide additional analysis in the following sections: Additional simulation methods describes details of the simulations including spatial discretization, approximations made, numerical stability, and the measure used for assessing improvements in robustness. Mechanistic interpretation of oscillatory model parameters formalizes the approach of using k_A and k_{AB} as limit-cycle coupled parameters. Dimensionality of parameter space discusses whether limit cycles can sufficiently improve Turing pattern formation in higher dimensions, and whether a high-dimensional parameter space can be compressed. Timescales of limit cycles provides SEF changes upon varying the angular speed of limit cycles. Properties of SEF provides additional analysis of SEF changes upon varying wavelength and amplitude of patterns, extending Fig. 2 in the main text. Interpretation of random search as spatial Poisson sampling process extends Fig. 2 with an analytical approach on intersection and encounter probabilities across repeats. Limit cycle and Turing island shapes on discovery of Turing islands supplements Fig. 3 by providing example Turing islands with highest and lowest intersection rates, and the distribution of the intersection and encounter probabilities. Variability of limit cycle and SEF complements Fig. 4, showing that reproducibility of limit-cycle models are dependent on the noise in the limit-cycle trajectory. Investigation of different dimensions addresses the question of limit cycles in 1D and 3D, in conjunction with Dimensionality of parameter space. Multistability within parameter space provides details of the discussion on multistability of our system. Fine tuning of French flag gradient demonstrates the requirement of fine-tuning of French-flag gradients for patterns that are strong and reproducible, extending Fig. 5. Details of oval limit cycles visualizes the distribution of r_x and r_y of oval limit cycles.

Additional simulation methods.

Here we provide some detailed methods, augmenting the main-text methods section.

Spatial discretization. We use a 2D finite-difference Laplacian, Eq. (1), to convert the PDEs in the reaction-diffusion system to a system of coupled ODEs. Let a denote the concentration of either molecular species, A or B, δr the grid spacing between each pixel, and D_a the diffusion constant; we write:

$$\nabla^2 a_{i,j} = D_a \frac{a_{i+1,j} + a_{i-1,j} + a_{i,j+1} + a_{i,j-1} - 4a_{i,j}}{\delta r^2} \quad [1]$$

This approach allows us to treat the PDE system as a matrix of ODEs in 2D space.

Approximation method. Spatial discretization and limit-cycle modeling make use of numerical approximation methods for the resulting dynamical system. To account for stochasticity, we consider multiplicative noise (1). The deterministic part of the dynamics is integrated using a second-order Runge–Kutta (RK2) scheme, while stochastic terms are incorporated using an Euler–Maruyama-type discretization.

Let x denote the variable of interest, which may represent k_1, k_2, A, or B, and let x_n denote its value at timestep n. The deterministic dynamics,

$$\frac{dx}{dt} = f(t, x), \quad [2]$$

are approximated using the predictor step

$$x_n^0 = x_n + f(t_n, x_n)\,\Delta t, \quad [3]$$

$$f^0 = f(t_{n+1}, x_n^0), \quad [4]$$

followed by the update

$$x_{n+1} = x_n + \frac{\Delta t}{2}\left(f(t_n, x_n) + f^0\right) + \sigma_x x_n\,\Delta W_n, \quad [5]$$

where $\Delta W_n \sim \mathcal{N}(0, \Delta t)$ represents the increment of a Wiener process.

For the limit-cycle variables k_1 and k_2, stochasticity is introduced separately through radial and tangential perturbations, rather than through the multiplicative noise term above.

Simulations were carried out in Python on the Imperial College High-Performance Computing cluster. We used no-flux boundary conditions, preventing movement of activator or inhibitor across the boundaries.

Numerical stability. The time step Δt of the simulation must be numerically stable, and it thereby needs to satisfy the condition (2):

$$\Delta t \leq \frac{\Delta x^2}{2dD_{max}} = 0.0025 \quad [6]$$

where $d = 2$ (dimensionality) and $\Delta x = 0.5$ (spatial grid). However, to be safe against the effect of stochastic noise that was implemented, we employ $\Delta t = 0.002$.

Measure of improvement. To assess the improvement in robustness through limit-cycle intersections, we computed the domain-size averaged difference between intersection and random-sampling encounter probabilities:

$$\Delta\bar{P}_{d,lc} = \frac{\sum_{i=1}^{n}\left(P_{d,lc}(r_i) - P_{d,rs}(r_i)\right)}{n}, \quad [7]$$

where d denotes dimensionality of the system, lc the type of limit cycle, rs random sampling, r_i the i-th domain radius, and n the total number of sampled radii.

Mechanistic interpretation of oscillatory model parameters.

In our Turing model, Eqs. 1 of the main text, k_A and k_{AB} are limit-cycle–coupled parameters representing half-saturation constants in the Hill functions and therefore setting the effective regulatory threshold of transcription factor binding. Such thresholds can be modulated dynamically in real regulatory networks. A well-known example is the mammalian circadian clock, where the transcription factors CLOCK and BMAL1 activate target genes such as *Per* and *Cry*, while the PER–CRY complex later suppresses CLOCK–BMAL1 activity and reduces its DNA binding capability (3). This delayed negative feedback generates a limit-cycle oscillation in transcription factor activity and thus an oscillatory effective promoter affinity. Downstream targets of CLOCK–BMAL1, such as Rev-Erbα, exhibit phase-shifted expression relative to CLOCK–BMAL1 and compete with other transcription factors for regulatory binding sites (4), providing an additional mechanism for oscillatory modulation of transcriptional thresholds.

To gain intuition, here we mechanistically model an oscillatory Hill threshold starting from competitive promoter binding. Consider a gene whose expression is activated by the transcription factor A (e.g. the CLOCK–BMAL1 heterodimer) and inhibited by a competing factor I (e.g. the PER–CRY complex). If the two regulators compete for promoter occupancy, the probability that the promoter is bound by the activator can be written as

$$f(A, I) = \frac{\frac{A}{k_A}}{1 + \frac{A}{k_A} + \frac{I}{k_I}}, \qquad [8]$$

where k_A and k_I represent effective binding constants for activator and inhibitor interactions, respectively. This expression can be rewritten in Hill form as

$$f(A, I) = \frac{\frac{A}{K_{\mathrm{eff}}(I)}}{1 + \frac{A}{K_{\mathrm{eff}}(I)}}, \qquad [9]$$

where the effective activation threshold is

$$K_{\mathrm{eff}}(I) = k_A \left(1 + \frac{I}{k_I}\right). \qquad [10]$$

Thus competitive inhibition shifts the activation threshold of the Hill function. Increasing concentrations of the inhibitor raise the effective threshold K_{eff}, meaning higher activator levels are required to achieve the same transcriptional output. If the inhibitor concentration oscillates in time, as occurs in circadian regulatory loops where PER–CRY periodically suppresses CLOCK–BMAL1 activity, the effective threshold $K_{\mathrm{eff}}(t)$ will also oscillate. In our coarse-grained gene regulatory model this corresponds to a periodic modulation of Hill parameters such as k_A or k_{AB}, providing a mechanistic interpretation of oscillatory threshold parameters.

Dimensionality of parameter space.

Here, we discuss applying limit cycles to different numbers of dimensions of the Turing parameter space. We first simulate a 1D system, with Turing region randomly located within the domain length. This eliminates the effects of limit-cycle and Turing-island shapes (Supplementary Fig. S9A). With each oscillator trying to discover Turing regions, intersection and encounter probabilities were significantly higher than random sampling (Supplementary Fig. S9B). These probabilities would be proportional to the amplitude of the oscillations, and the size of the Turing regions.

We also investigated 3D limit cycles with randomly shaped Turing volumes randomly located within a sphere (Supplementary Fig. S10A,B). We observe a reduction in the random-sampling encounter probability (Supplementary Fig. S10C). This reduction results in limit cycles having encounter probabilities higher than those of random sampling. We also observe a general reduction in intersection probability compared to the 2D system (Supplementary Fig. S10D). These trends are intuitive considering the rotational freedom and free space that higher dimensions provide. As visualized in Supplementary Fig. S10E, shapes with higher sphericity tend to have lower intersection probabilities, but the reverse can also be true.

The question remains whether the 11 parameters of our model can be effectively reduced to lower dimensions (5), allowing for the improvement of the discovery of the Turing island. To identify the combinations of parameters that most strongly influence pattern formation, we performed a local sensitivity analysis of the dispersion relation following the framework developed for sloppy models (6, 7).

We consider a reaction–diffusion system

$$\partial_t \mathbf{u} = \mathbf{f}(\mathbf{u}; \mathbf{p}) + D\nabla^2 \mathbf{u}, \quad [11]$$

where $\mathbf{u}$ denotes the vector of species concentrations, $\mathbf{p}$ the model parameters, and D the diffusion matrix. Let $\mathbf{u}_0(\mathbf{p})$ denote the homogeneous steady state satisfying

$$\mathbf{f}(\mathbf{u}_0; \mathbf{p}) = 0. \quad [12]$$

Linearising the dynamics around this steady state and considering perturbations of the form

$$\delta\mathbf{u} \propto e^{\lambda(q)t + iqx}, \quad [13]$$

leads to the dispersion relation

$$\lambda(q) = \max \mathrm{Re}\left[\mathrm{eig}\left(J - q^2 D\right)\right], \quad [14]$$

where

$$J = \frac{\partial \mathbf{f}}{\partial \mathbf{u}}\bigg|_{\mathbf{u}_0} \quad [15]$$

is the Jacobian of the reaction kinetics evaluated at the steady state.

To quantify how the dispersion relation depends on the parameters, we evaluated $\lambda(q)$ on a discrete set of wavenumbers $\{q_i\}$ and defined the observable vector

$$\mathbf{y}(\mathbf{p}) = [\lambda(q_1; \mathbf{p}), \lambda(q_2; \mathbf{p}), \ldots, \lambda(q_N; \mathbf{p})]. \quad [16]$$

Sensitivities were computed with respect to logarithmic parameter perturbations,

$$S_{ij} = \frac{\partial y_i}{\partial \log p_j}, \quad [17]$$

which correspond to multiplicative changes in parameter values and ensure that sensitivities are dimensionless and comparable across parameters with different scales. The derivatives were evaluated numerically using finite differences.

From the sensitivity matrix S we construct the Fisher information matrix

$$F = S^\top S. \quad [18]$$

The eigenvalues of F quantify the sensitivity of the dispersion relation to orthogonal directions in parameter space. Large eigenvalues correspond to *stiff* parameter combinations that strongly modify the dispersion relation, whereas small eigenvalues correspond to *sloppy* directions along which parameter perturbations have little effect on the behaviour of the system. By plotting the contributions of each parameter to each eigenvector (Supplementary Fig. S1A), we observe that most parameters are involved in the stiffest directions. We can also see that the scale of the eigenvalues of the Fisher information matrix falls sharply after the fifth eigenvalue (Supplementary Fig. S1B).

To facilitate comparison between parameter sets, eigenvalues were normalized by the largest eigenvalue,

$$\tilde{\lambda}_k = \frac{\lambda_k}{\lambda_1}. \quad [19]$$

To assess the robustness of the resulting spectra, the analysis was repeated for multiplicatively perturbed parameter sets in the vicinity of the reference point. Only parameter sets that preserved a proper Turing instability were retained in the analysis. We then plotted the relative magnitude of the eigenvalues resulting from the perturbed parameters and found that in most cases, there are 5-6 stiff directions, and the rest are sloppy (Supplementary Fig. S1C).

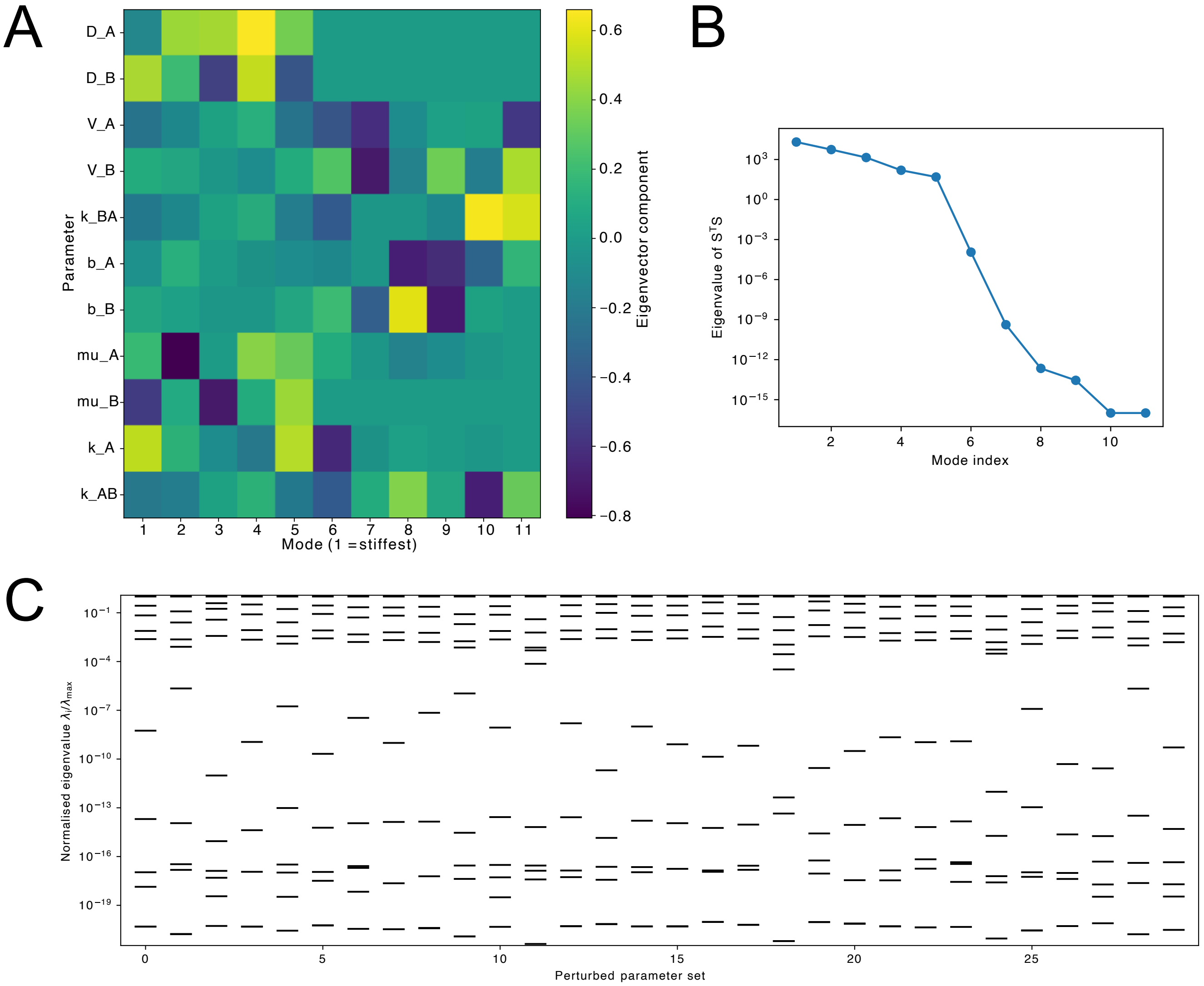

Supplementary figure S1. Sensitivity analysis of the dispersion relationship. (A) Magnitude of each parameter in the eigenvectors ordered by stiffness. **(B)** Eigenvalues of the Fisher information matrix. We can observe that there is a sudden drop after the top 5 eigenvalues, signifying that those are associated with the stiffest directions. **(C)** Normalised eigenvalues of the Fisher information matrix for a perturbation around the initial parameter set. We can observe that the drop after 5–6 eigenvalues occurs in most cases, so it is not a parameter-specific phenomenon.

Timescales of limit cycles.

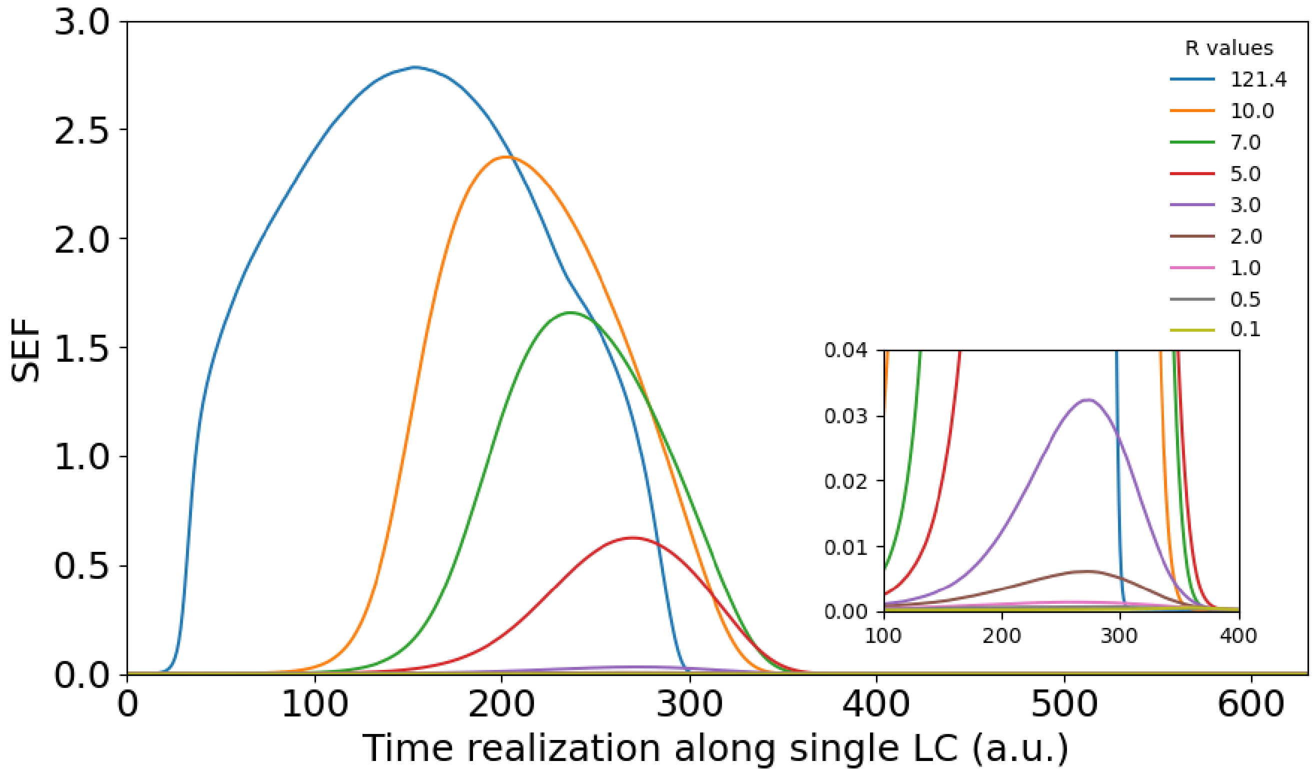

Supplementary figure S2. Effect of varying velocity on pattern formation. Sensitivity measure $R = \lambda s/(\omega r)$ is used, where λ corresponds to the mean of maximum eigenvalue along the arclength s, ω denotes the angular velocity, and r is the radius of the limit cycle. The R value is controlled through varying ω. The center of the limit cycle is set to be same as circle 6 of Fig. 4. Hence, $r = 5$, $\lambda =$ 0.430 and $s = 2\pi r \times 0.45$ for this limit cycle. The time has been normalized so that the parameters travel around the limit cycle only once for its given angular speed. This results in the fixed lag time being represented as longer as we move into smaller time domains.

Properties of SEF.

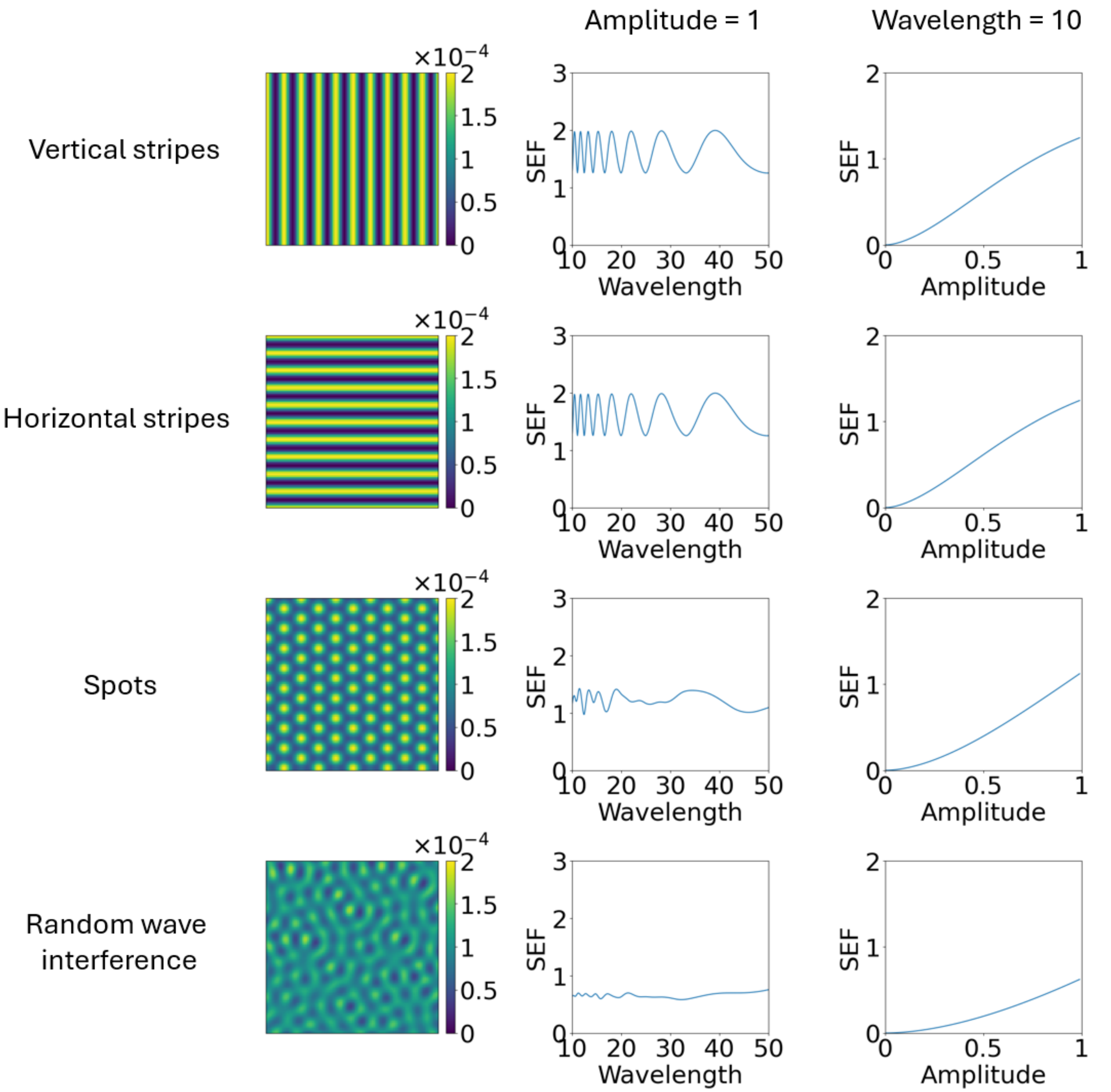

Supplementary figure S3. Visualization of patterns of different amplitude and orientation with corresponding SEF values. Stripes were constructed through: $1 + A\cos(\frac{2\pi x}{\lambda})$ (replace x with y for horizontal stripes), spots by: $1 + \frac{A}{3}\sum_{i=1}^{3}\cos(\frac{2\pi}{\lambda}(\alpha_i x + \beta_i y))$, where $\alpha = [1, -0.5, -0.5]$ and $\beta = [0, \frac{\sqrt{3}}{2}, -\frac{\sqrt{3}}{2}]$. Random wave interference through: $1 + \frac{A}{9}\sum_{i=1}^{9}\cos(\frac{2\pi}{\lambda}(x\cos(\theta_i) + y\sin(\theta_i)) + \phi_i)$ where $\theta_i \sim U(0, 2\pi)$ and $\phi_i \sim U(0, 2\pi)$. A denotes amplitude and λ denotes wavelength.

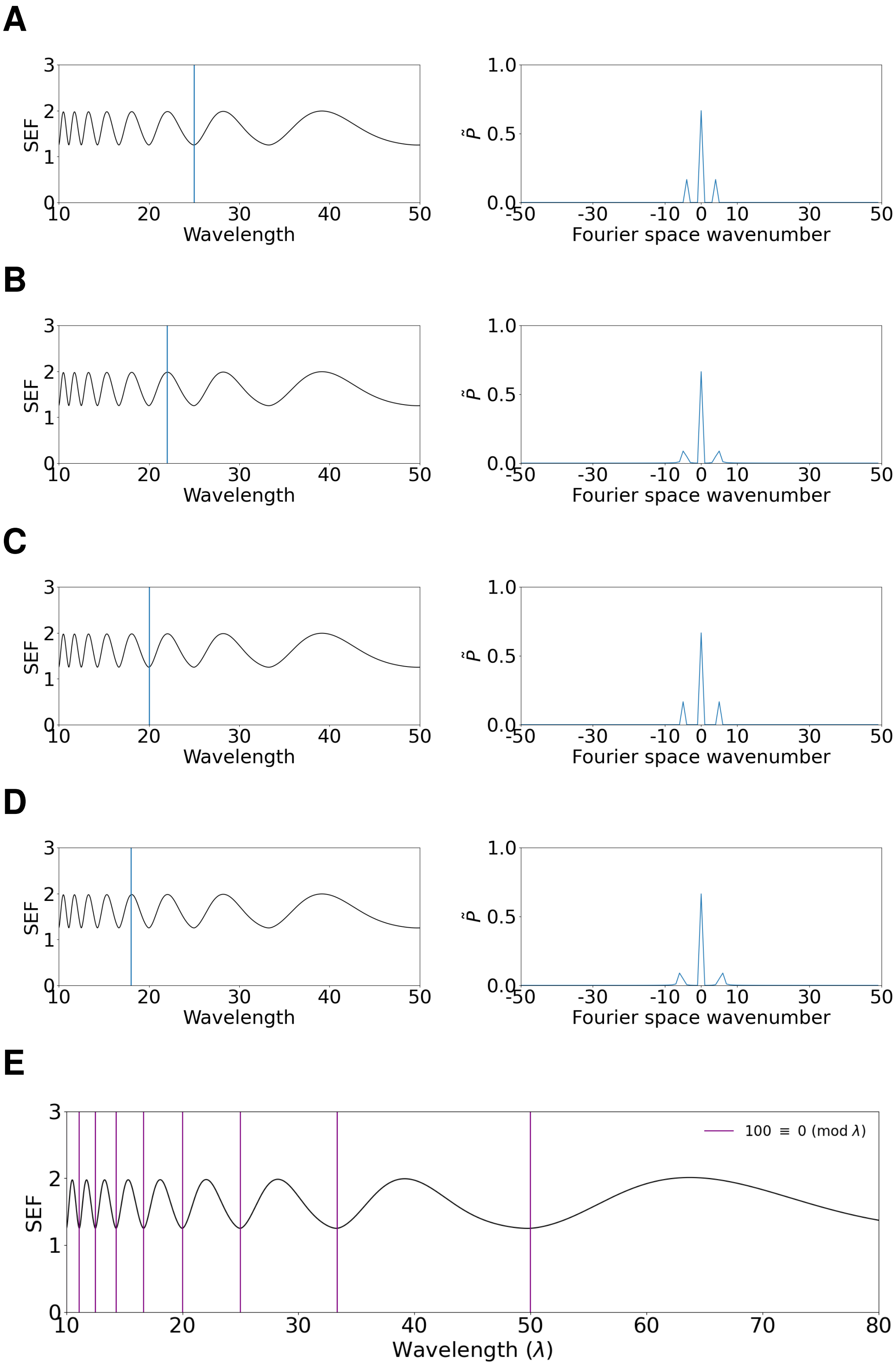

Supplementary figure S4. Explanation of the oscillatory SEF values across different wavelengths. We visualize the normalized power spectra of real-space cosine waves with wavelengths **(A)** 25, **(B)** 22, **(C)** 20 and **(D)** 18. The results show that waves with wavelengths that are divisible by 100 (size of the grid) tend to show lowest SEF values, due to a sharper distribution in Fourier space compared to waves that cannot. On the other hand, wavelengths that are distant from either whole number dividers tend to produce high SEF values. We visualize this in **(E)** with SEF values between wavelengths of 10 to 80. Purple lines indicate wavelength values that divide 100 (space size) perfectly, resulting in low SEF. While there are infinite number of wavelengths capable of such division, for our 100×100 space, the relevant wavelengths are between 10 and 50.

 Seyoon Kim, Antonio Matas-Gil, and Robert G. Endres

Interpretation of random search as spatial Poisson sampling process.

By fixing the radius of the limit cycle and approximating the trajectory as a circumference, we treat the search as a spatial Poisson process over possible positions of the cycle center. For ease of visualization, we assume that the Turing region, the limit cycle and the overall search domain are circular, with radii R_1, R_2 and R_3, respectively, as depicted in Supplementary Fig. S5.

Under a standard random sampling strategy, the probability of encountering the Turing region is simply the ratio of their areas. Upon canceling factors of π, we obtain

$$P_E^{RS} = \left(\frac{R_1}{R_3}\right)^2,$$

with $R_1 < R_3$. In contrast, for the limit-cycle strategy we fix the cycle radius R_2 (with $R_2 > R_1$) and consider which positions of the cycle center lead to an intersection between the cycle and the Turing region. We have two different possibilities here. First, if $R_1 < R_2$, an encounter occurs whenever the center of the limit cycle lies at a distance between $R_2 - R_1$ and $R_2 + R_1$ from the center of the Turing region. The set of such centers therefore forms an annulus with inner radius $R_2 - R_1$ and outer radius $R_2 + R_1$. The corresponding intersection probability is:

$$P_F^{LC} = \frac{(R_2 + R_1)^2 - (R_2 - R_1)^2}{R_3^2} = \frac{4R_2R_1}{R_3^2}.$$

This shows that whenever $R_2 > R_1/4$, the limit-cycle strategy outperforms the random point-search strategy (Supplementary Fig. S5A). Moreover, the advantage increases with R_2, although trajectories intersecting the Turing region will tend to spend a smaller fraction of their total path inside it. On the other hand, if $R_2 \leq R_1$, any limit cycle with center inside the Turing region will intercept the Turing region (except the actual center if $R_2 = R_1$), and there will be an extra region around the Turing region which will also lead to intersection (Supplementary Fig. S5B). Summing both areas leads to a circle of radius $R_1 + R_2$. Hence, the intersection probability in this case is

$$P_F^{LC} = \frac{(R_2 + R_1)^2}{R_3^2}$$

The ratio between the encounter probability of random search and the intersection probability of limit cycles for $R_1 < R_2$ is

$$\frac{P_F^{LC}}{P_E^{RS}} = \frac{4R_2}{R_1},$$

showing that the improvement grows linearly with R_2. For the case $R_1 > R_2$, we obtain:

$$\frac{P_F^{LC}}{P_E^{RS}} = \frac{(R_2 + R_1)^2}{R_1^2} = \left(1 + \frac{R_2}{R_1}\right)^2.$$

Hence, the improvement grows quadratically at first, until it gets to $R_1 < R_2$ and starts growing linearly.

To consider the ratio of the encounter probabilities, we need to compute the ratio between the region that intercepts the limit cycle and the whole limit cycle. We can write this ratio as a function of the distance to the Turing region center:

$$P_E^{LC}(d) = \frac{2R_2 \arccos\left(\frac{d^2 + R_2^2 - R_1^2}{2dR_2}\right)}{2\pi R_2} = \frac{1}{\pi} \arccos\left(\frac{d^2 + R_2^2 - R_1^2}{2dR_2}\right) \quad [20]$$

To obtain the encounter probability, we would need to integrate this quantity (multiplied by $2\pi d$) from $d = R_2 - R_1$ to $d = R_2 + R_1$:

$$P_E^{LC} = \int_{d=R_2-R_1}^{d=R_2+R_1} 2d \arccos\left(\frac{d^2 + R_2^2 - R_1^2}{2dR_2}\right) \mathrm{d}d = \pi R_1^2 \quad [21]$$

This last simplification follows from the fact that $2\arccos\left(\frac{d^2+R_2^2-R_1^2}{2dR_2}\right)$ is the angular span of the portion of the circle of radius R_2 that lies inside the circle of radius R_1. Hence, we find that the limit-cycle strategy has the same encounter probability as random search, but a higher intersection probability.

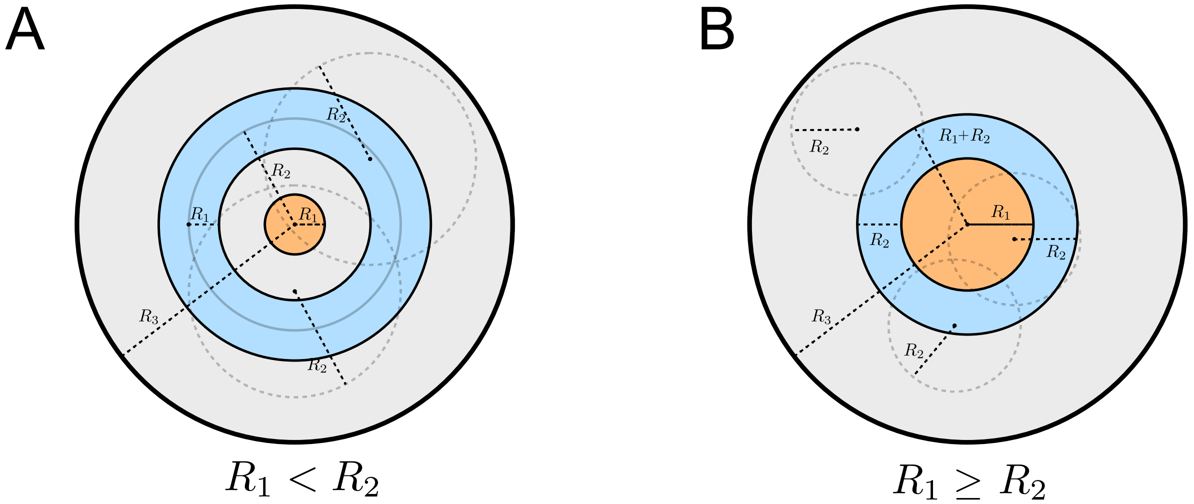

Supplementary figure S5. Poisson sampling interpretation of the limit cycle strategy for (A) $R_1 < R_2$ and (B) $R_1 \geq R_2$. The orange region depicts the Turing region of radius R_1. The blue region depicts the centers whose limit cycles will intercept the Turing region. Dashed circles represent different limit cycles of radius R_2. The whole search area is shown as a circle of radius R_3. When $R_1 < R_2$ (A), we can observe that the blue area forms an annulus of outer radius $R_1 + R_2$ and inner radius $R_2 - R_1$, whereas if $R_1 > R_2$ (B), the blue area encompasses all of the Turing region, plus a small ring just outside of the Turing region of diameter R_2.

Limit cycle and Turing island shapes on discovery of Turing islands.

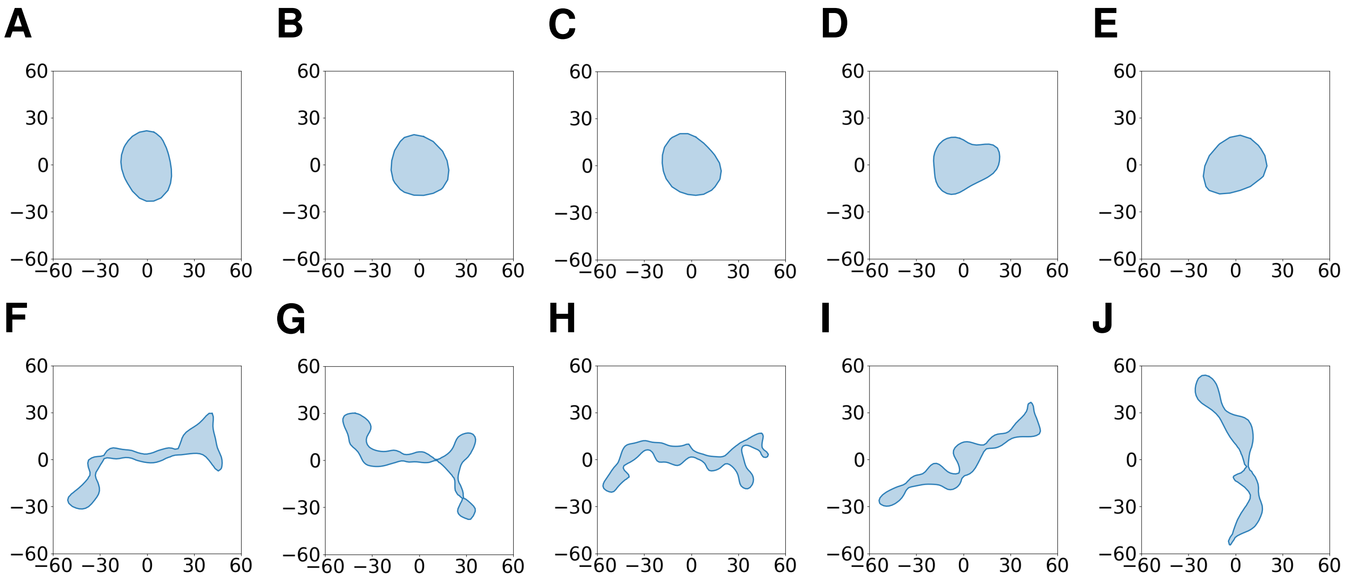

Supplementary figure S6. Example Turing island shapes with lowest and highest circle intersection probability. Lowest intersection probabilities: **(A)** 0.1195, **(B)** 0.1199, **(C)** 0.1206, **(D)** 0.1208, **(E)** 0.1212. Highest intersection probabilities **(F)** 0.2523, **(G)** 0.2499, **(H)** 0.2425, **(I)** 0.2419, **(J)** 0.2406.

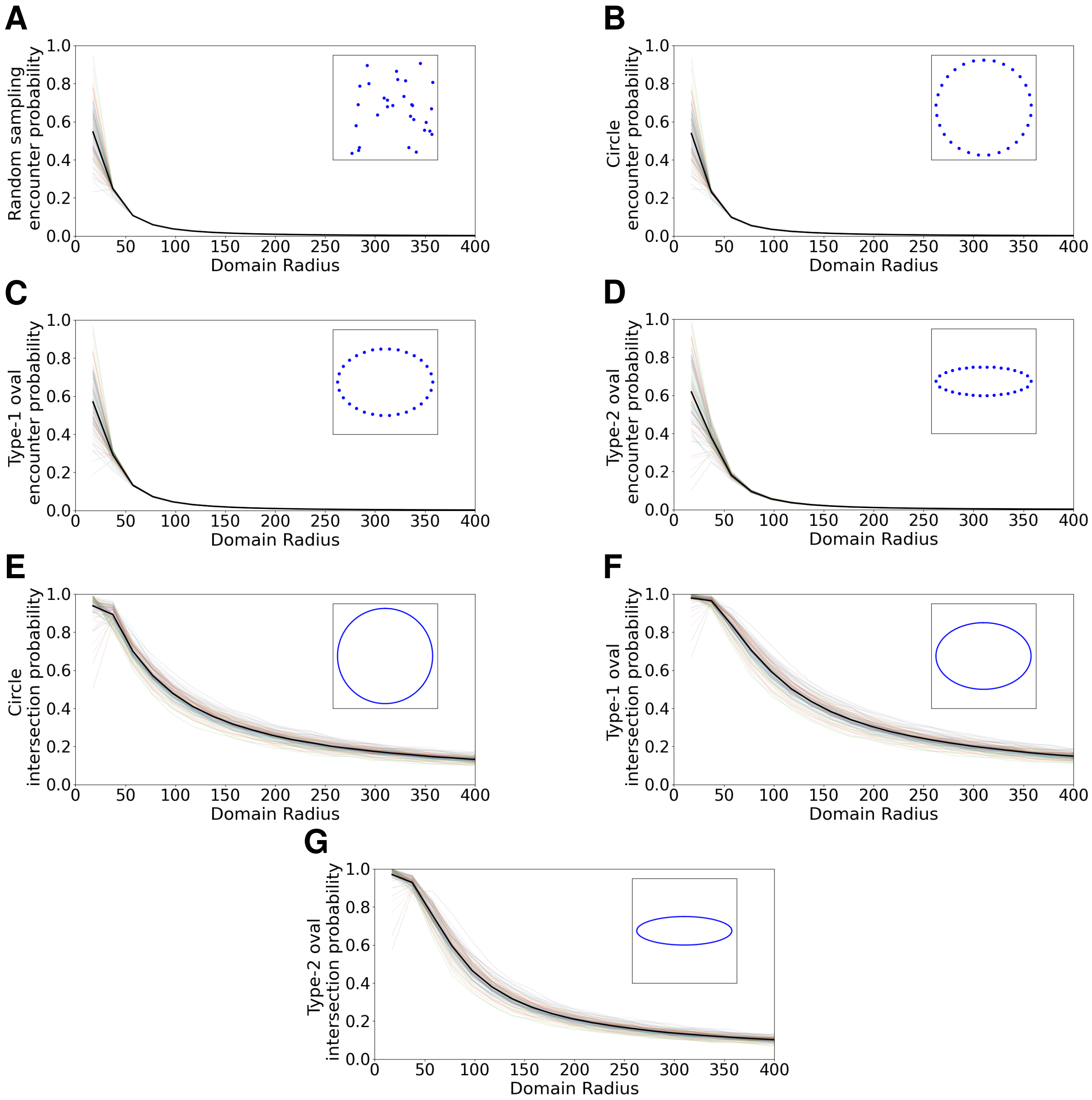

Supplementary figure S7. Visualization of respective intersection/encounter probabilities for 100 random Turing islands. (A) Random sampling encounter, **(B)** circle encounter, **(C)** type-1 oval encounter, **(D)** type-2 oval encounter, **(E)** circle intersection, **(F)** type-1 oval intersection and **(G)** type-2 oval intersection.

Variability of limit cycle and SEF.

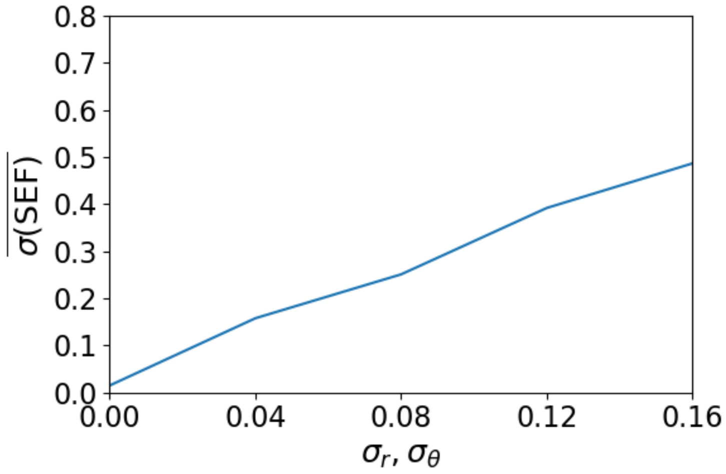

Supplementary figure S8. Limit cycle noise and SEF standard deviation. Change in mean of SEF standard deviation in limit cycle 6 with corresponding noise across 8 repeats. Radial and tangential noise were set equal.

Investigation of different dimensions.

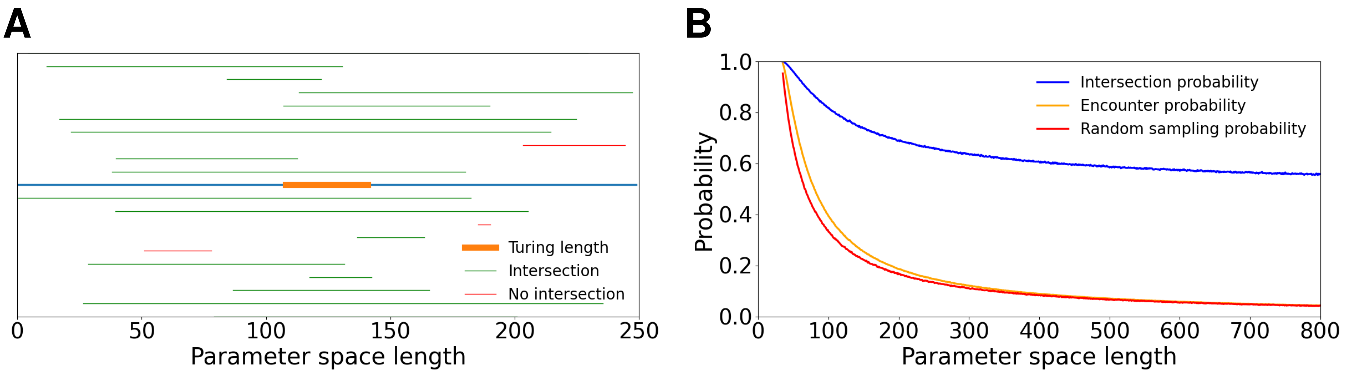

Supplementary figure S9. 1D Turing regions and limit-cycle oscillations. **(A)** Schematic of 1D Turing-segment simulation, displaying limit cycles alongside Turing segments in orange. Green lines represent limit cycles that encountered Turing regions, and red line represent those that did not. **(B)** The encounter and intersection probabilities of the 1D Turing and limit-cycle setup. The Turing region was set to have length $\sqrt{1112} \approx 33.3$.

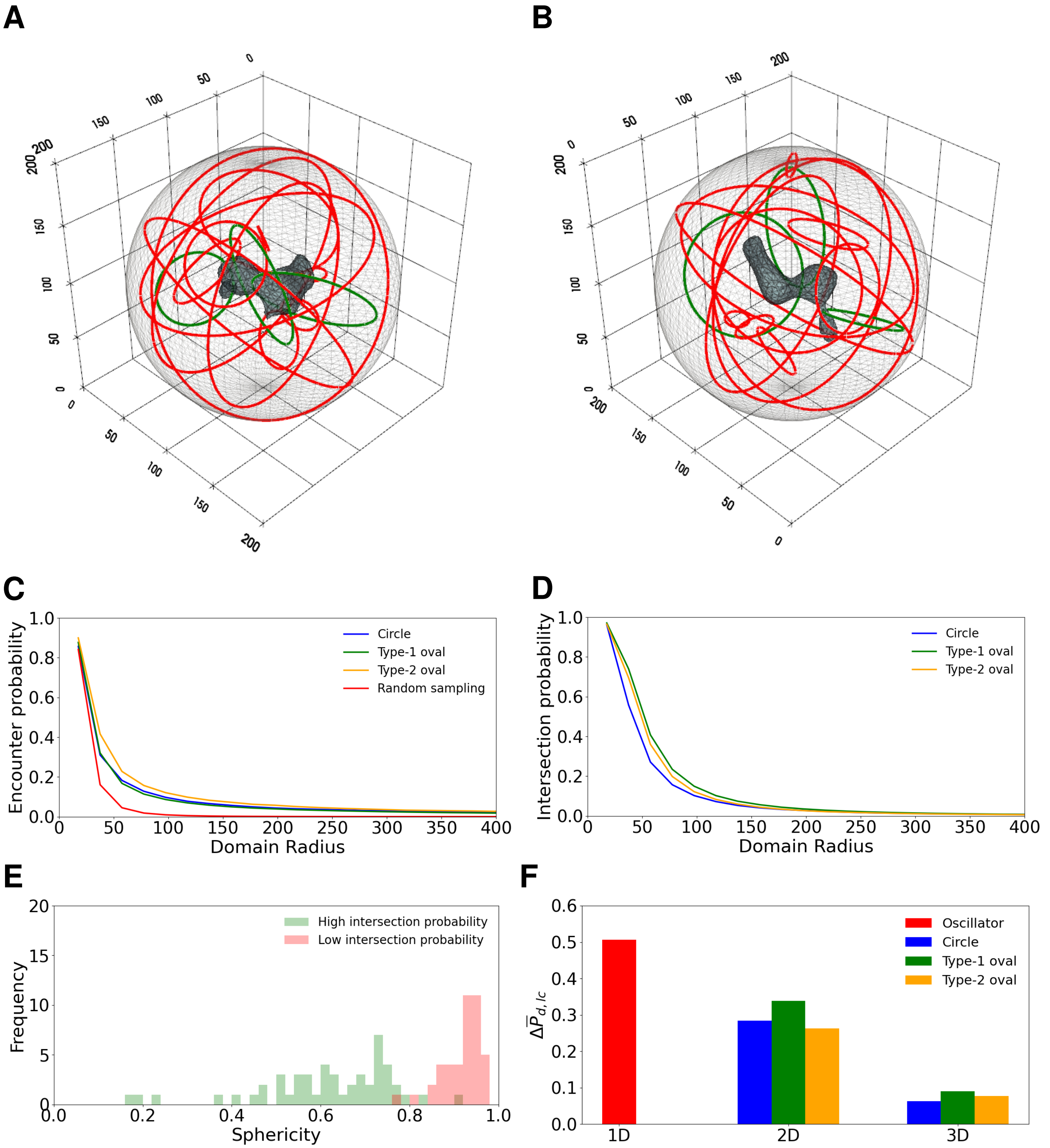

Supplementary figure S10. 3D Turing volume and limit cycles. (A, B) Visualization of circular limit cycles and Turing region in 3D space shown from opposite perspectives around the z axis. Turing regions were set to have volume of $1112\sqrt{1112} \approx 37081.5$. **(C)** Encounter probability of different limit cycles and random sampling to randomly shaped 3D Turing island. **(D)** Intersection probability of different limit cycles to randomly shaped 3D Turing island. **(E)** Distribution of shape sphericity for lowest and highest circle intersection probability. **(F)** Difference between intersection probability and random sampling encounter probability, averaged over domain radius for each dimension (Eq. (7)).

Multistability within parameter space.

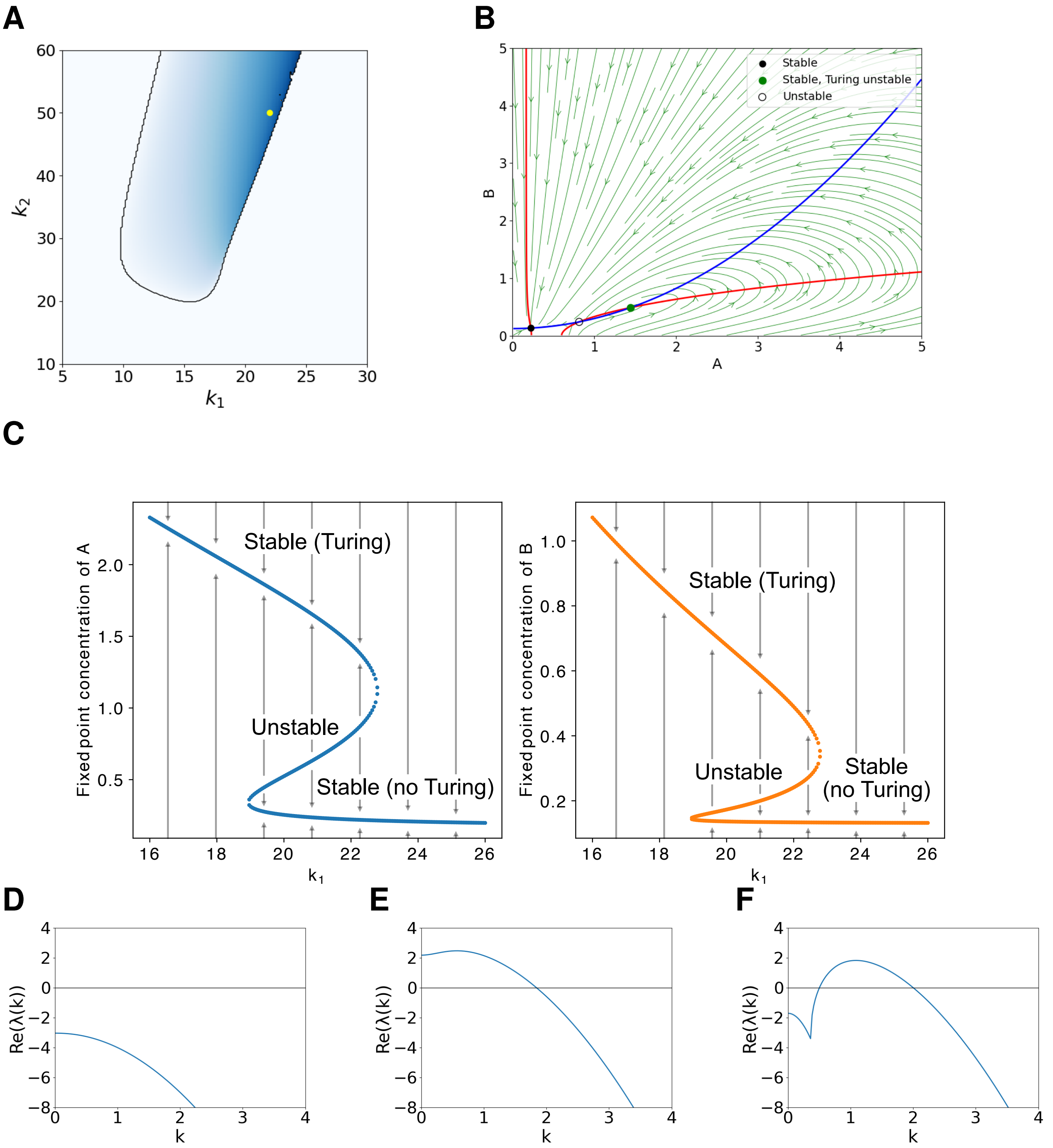

Supplementary figure S11. Multistability within parameter space. (A) Visualization of parameter space with yellow dot at $k_1 = 22$, $k_2 = 50$. **(B)** Phase diagram illustrating the three steady states at $k_1 = 22$, $k_2 = 50$. The green arrows indicate the direction of derivatives at respective concentration of activator A and inhibitor B. **(C)** Bifurcation diagram of the system. We fixed $k_2 = 50$ and vary k_1 between 10 and 30. We observe that for small values of k_1 we have a single fixed point which is stable and produces Turing patterns with diffusion turned on. At $k_1 \approx 19$, we observe a supercritical saddle-node bifurcation, where two new fixed points are created, one unstable and another stable. This stable one does not produce Turing patterns. At $k_1 \approx 23$, a subcritical saddle-node bifurcation between the first stable point and the unstable point occurs, leaving only the second stable point, which continues to not produce Turing patterns. **(D-F)** Dispersion relations of stable, unstable and Turing systems.

Fine tuning of French flag gradient.

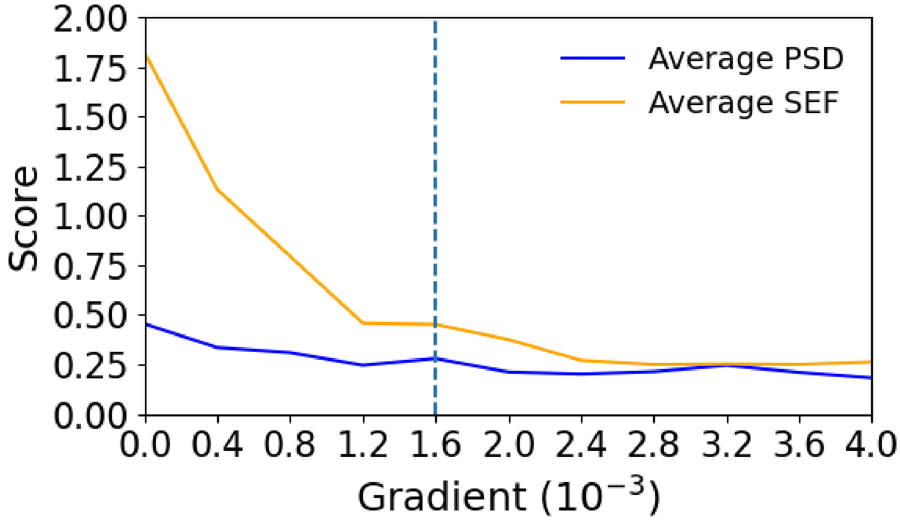

Supplementary figure S12. Selection of French-flag gradient. Change in average PSD and SEF over varying French flag gradient. The blue dotted line represents the gradient employed in our model. Higher strength of gradient reduces PSD, indicating increase in reproducibility but in the cost of patterns (SEF). As the fluctuations in PSD due to limit cycle noise exceeds the improvements by the gradient more easily if the overlap with Turing island is small, it is hard to tune the gradient using limit cycles with low intersection. Therefore, we used Limit cycle 8, which has a high intersection with Turing island, to tune our gradient.

Details of oval limit cycles.

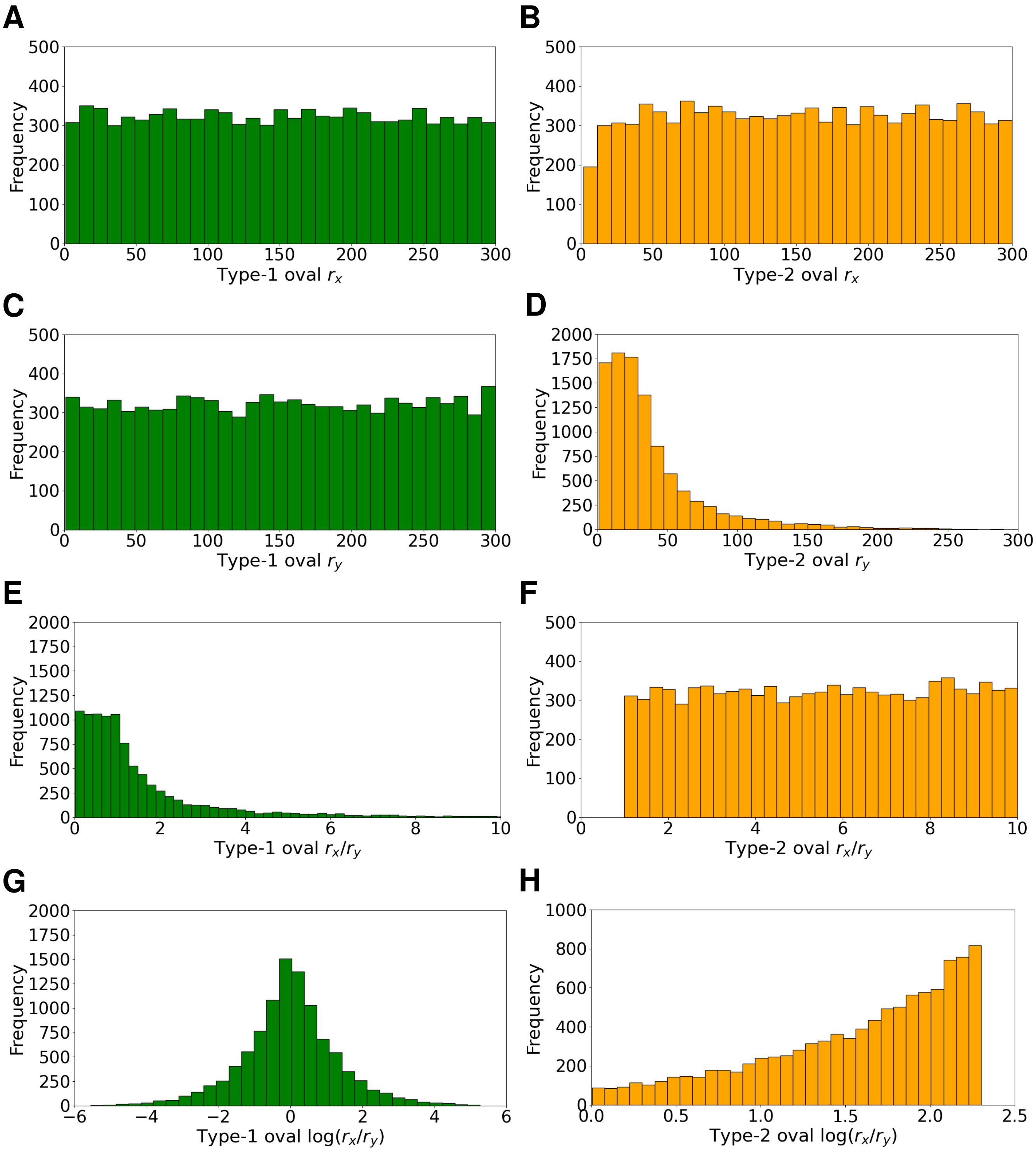

Supplementary figure S13. Histograms of 10^4 r_x and r_y pairs and their ratios for type-1 and type-2 ovals, with $r_{max} = 300$ a.u. (A, B) Distributions of r_x for type-1 and type-2 ovals. **(C, D)** Distribution of r_y. **(E, F)** Distributions of r_x/r_y. **(G, H)** Distributions of $\log(r_x/r_y)$ to reflect the full distribution of r_x/r_y. The type-1 ovals are focused on uniform distribution of r_x and r_y, resulting in low frequency of ovals with extreme r_x/r_y values. The type-2 ovals are instead focused on uniform distribution of r_x/r_y, with a higher frequency of r_x/r_y extremes.

References

1. J Raspopovic, L Marcon, L Russo, J Sharpe, Digit patterning is controlled by a Bmp-Sox9-Wnt Turing network modulated by morphogen gradients. *Science* **345**, 566–570 (2014).
2. P Moin, ed., NUMERICAL SOLUTION OF PARTIAL DIFFERENTIAL EQUATIONS in *Fundamentals of Engineering Numerical Analysis.* (Cambridge University Press, Cambridge), 2 edition, pp. 101–166 (2010).
3. Y Otobe, et al., Phosphorylation of DNA-binding domains of CLOCK–BMAL1 complex for PER-dependent inhibition in circadian clock of mammalian cells. *Proc. Natl. Acad. Sci.* **121**, e2316858121 (2024).
4. H Cho, et al., Regulation of Circadian Behavior and Metabolism by Rev-erbα and Rev-erbβ. *Nature* **485**, 123–127 (2012).
5. BB Machta, R Chachra, MK Transtrum, JP Sethna, Parameter Space Compression Underlies Emergent Theories and Predictive Models. *Science* **342**, 604–607 (2013).
6. RN Gutenkunst, et al., Universally sloppy parameter sensitivities in systems biology models. *PLoS computational biology* **3**, 1871–1878 (2007).
7. KS Brown, JP Sethna, Statistical mechanical approaches to models with many poorly known parameters. *Phys. Rev. E* **68**, 021904 (2003).
